# A miRNA Signature for Cognitive Deficits and Alcohol Use Disorder in Persons Living with HIV/AIDS

**DOI:** 10.3389/fnmol.2017.00385

**Published:** 2017-11-15

**Authors:** Dorota Wyczechowska, Hui-Yi Lin, Andrea LaPlante, Duane Jeansonne, Adam Lassak, Christopher H. Parsons, Patricia E. Molina, Francesca Peruzzi

**Affiliations:** ^1^Stanley S. Scott Cancer Center, Louisiana State University, New Orleans, LA, United States; ^2^Biostatistics Program, School of Public Health, Louisiana State University, New Orleans, LA, United States; ^3^Department of Psychiatry, University Medical Center, Louisiana State University, New Orleans, LA, United States; ^4^Stanley S. Scott Cancer Center, Department of Medicine, School of Medicine, Louisiana State University, New Orleans, LA, United States; ^5^Alcohol and Drug Abuse Center of Excellence, Department of Physiology, School of Medicine, Louisiana State University, New Orleans, LA, United States; ^6^Stanley S. Scott Cancer Center, Alcohol and Drug Abuse Center of Excellence, Department of Medicine, School of Medicine, Louisiana State University, New Orleans, LA, United States

**Keywords:** HIV, HIV-associated neurocognitive disorders, miRNA, biomarker, substance abuse, alcohol use disorder

## Abstract

HIV-associated neurocognitive disorders (HAND) affects more than half of persons living with HIV-1/AIDS (PLWHA). Identification of biomarkers representing the cognitive status of PLWHA is a critical step for implementation of successful cognitive, behavioral and pharmacological strategies to prevent onset and progression of HAND. However, the presence of co-morbidity factors in PLWHA, the most common being substance abuse, can prevent the identification of such biomarkers. We have optimized a protocol to profile plasma miRNAs using quantitative RT-qPCR and found a miRNA signature with very good discriminatory ability to distinguish PLWHA with cognitive impairment from those without cognitive impairment. Here, we have evaluated this miRNA signature in PLWHA with alcohol use disorder (AUD) at LSU Health Sciences Center (LSUHSC). The results show that AUD is a potential confounding factor for the miRNAs associated with cognitive impairment in PLWHA. Furthermore, we have investigated the miRNA signature associated with cognitive impairment in an independent cohort of PLWHA using plasma samples from the CNS HIV Antiretroviral Therapy Effects Research (CHARTER) program. Despite differences between the two cohorts in socioeconomic status, AUD, and likely misuse of illicit or prescription drugs, we validated a miRNA signature for cognitive deficits found at LSUHSC in the CHARTER samples.

## Introduction

Neurocognitive decline affects daily life of more than half of persons living with HIV/AIDS (PLWHA). Identification of patients at risk of developing neurocognitive impairment would allow for early diagnosis and interventions; however, to date there are no biomarkers available in the clinical setting. There are three major types of HIV-associated neurocognitive disorders (HAND): (1) asymptomatic neurocognitive impairment (ANI), which is assessed by cognitive testing but is not clinically evident; (2) mild neurocognitive disorders (MND), is diagnosed by exclusion and involves mild functional impairment; (3) HIV-associated dementia (HAD) when moderate to severe impairment is clinically manifested. While the combined anti-retroviral therapy (cART) greatly improved life expectancy and reduced the prevalence of severe dementia, it did not improve HAND prevalence, which remains at about 50% (McArthur et al., 1993; Heaton et al., 2010). Diagnosis of MND, the most common form of HAND in PLWHA on cART, is assessed upon manifestation of symptoms, through standardized neuropsychological testing, imaging and exclusion of other causes of cognitive dysfunction (Antinori et al., 2007; Schouten et al., 2011; Saylor et al., 2016). PLWHA often suffer from a variety of comorbidities including hepatitis C virus (HCV) co-infection, metabolic and vascular disorders, as well as depression and Alzheimer’s disease (AD), although the incidence of AD in the HIV-population does not seem to be higher than in HIV-negative individuals (Ances et al., 2010; Ortega and Ances, 2014). Another important factor related to the distress of living with a debilitating chronic disease such as HIV is suicidal behavior. Suicidal ideation is relatively common among HIV patients and appears to be associated with a variety of factors, including major depression and substance abuse (Kalichman et al., 2000; Komiti et al., 2001; Wilcox et al., 2004; Carrico et al., 2006, 2007). The presence of behavioral (suicidal behavior and use of illicit substances) and socio-economic factors (e.g., poverty and education) add a degree of complexity challenging the identification of biomarkers for neurocognitive decline in this patient population. Despite this complexity, the development of new technologies allowed for the search of biomarkers, a research that has heavily intensified in the past few years.

MicroRNAs (miRNAs) are abundantly expressed in the brain (Narayan et al., 2015), where they regulate synaptic plasticity (Hu and Li, 2017) and brain development (Fagiolini et al., 2009; Ziats and Rennert, 2014; Chen and Qin, 2015; Davis et al., 2015), implying that miRNA dysregulation may parallel neurocognitive dysfunction. The role of miRNAs in neurodegenerative disorders is amply documented (Sun et al., 2015; Su et al., 2016; Harrison et al., 2017; Salta and De Strooper, 2017; Saraiva et al., 2017) and their role in mental health is rapidly emerging (Serafini et al., 2014; Lai et al., 2016; Banach et al., 2017; Fiori et al., 2017; Narahari et al., 2017; Olde Loohuis et al., 2017). Due to their high stability in body fluids, miRNAs have been considered as potential biomarkers for a variety for pathologies including neurodegenerative and psychiatric disorders (Kolshus et al., 2014; Maffioletti et al., 2016; Choi et al., 2017; Narahari et al., 2017). We have focused on plasma miRNAs and identified a plasma miRNA signature associated with neurocognitive impairment in a cohort of PLWHA in care at LSU Health Sciences Center (LSUHSC) HIV Outpatient Clinic (HOP; Kadri et al., 2015). In the current study, we have found a miRNA signature (miR-143-3p, miR-199b-5p and the combinations of miR-143-3p with miR-146a-5p, miR-485-3p, miR-126-5p and miR-484-3p) associated with alcohol use disorder (AUD) measured using the alcohol use disorder identification test (AUDIT) at LSUHSC. In addition, we found that AUD is a potential confounding factor for the miRNAs associated with cognitive impairment in HIV-patients. We have validated a miRNA signature of 9 miRNAs (miR-126-5p, miR-143-3p, miR-337-3p, miR-377-3p, miR-376a-3p, miR-495-3p, miR-127-3p, miR-197-3p and miR-194-5p) discriminating people with cognitive impairment from those without in a different cohort of plasma samples collected through the CNS HIV Antiretroviral Therapy Effects Research (CHARTER) program (Heaton et al., 2010). Finally, through our robust method we found 15 miRNA pairs able to distinguish cognitively impaired (CI) PLWHA at both LSUHSC and CHARTER.

## Materials and Methods

### Whole Blood Collection

Following informed written consent, 30 ml whole blood was collected from PLWHA at the Louisiana State University Health Sciences Center (LSUHSC) HOP Clinic in New Orleans, Louisiana in the context of routine health assessment visits to this clinic. Samples were de-identified using an alphanumeric coding system, and whole blood was transported twice daily (within 2 h of collection) to the HIV Specimen Biorepository housed within the Stanley S. Scott Cancer Center (SSSCC) where plasma was immediately separated from cell fractions and stored at −80°C. Patients with opportunistic infections involving the CNS, existing CNS tumors, history of significant head injury, multiple sclerosis, and other dementing disorders (AD) were not enrolled in the study. Details on demographic and relevant clinical parameters are shown in Table 1.

**Table 1:** Demographics and other parameters of patients at LSU Health Sciences Center (LSUHSC).

		CI (*n* = 34)	nonCI (*n* = 32)
Age	Avg	50.9	50.5
	Min–Max	40–60	41–66
Gender	Males (59%)	20	19
	Females (41%)	14	13
Ethnicity	African-American (89.5%)	29	30
	Caucasian (9%)	4	2
	Multi/Hispanic (1.5%)	1/1	0/0
Education	≤8 (14%)	7	2
	>8	27	30
GDS		≥0.5	<0.5
AUD	<8 (*n* = 39)	22 (12 M, 10 F)	17 (11 M, 6 F)
	≥8 (*n* = 27)	12 (8 M, 4 F)	15 (8 M, 7 F)
VL	Avg	10006	41275
	Undetected	12	8
CD4	Avg	486.5	372

### CHARTER Samples

General characteristics of the CHARTER study are reported elsewhere (Heaton et al., 2010). The details on demographic and some of the clinical parameters for the plasma samples used in this study are shown in Table 2.

**Table 2:** Demographics and other parameters of patients from the CNS HIV Antiretroviral Therapy Effects Research (CHARTER) study.

		CI (*n* = 35)	nonCI (*n* = 35)
Age	Avg	48.54	48.94
	Min–Max	45–54	41–54
Gender	Males (81.4%)	29	28
	Females (18.6%)	6	7
Ethnicity	African-American (55.7%)	13	26
	Caucasian (28.6%)	12	8
	Hispanic (12.8%)	8	1
Education	≤8	0	0
	>8	35	35
GDS		≥0.5	<0.5
AUD	<8	35	35
	≥8	0	0
BMI	<25	14	15
	25–30	11	8
	>30	8	10
VL	Avg	18820	35180
CD4	Avg	400	541
HCV		15	19

### Cognitive Testing

Cognitive functioning was assessed using a modified version of the Multicenter AIDS Cohort Study (MACS) screening battery, which measures memory, attention, processing speed, language, and motor skill. Participants completed six measures of cognitive functioning: WAIS-IV Digit Span, Controlled Oral Word Association Test, Rey Auditory Verbal Learning Test, Trail Making Test, Symbol Digit Modalities Test and Grooved Pegboard Test. Raw scores on these measures were converted to t-scores using demographically-adjusted normative data. T-scores were converted to deficit scores and averaged to develop Global Deficit Scores (GDS) using methods previously reported by Heaton et al. (1995) and Carey et al. (2004). GDS scores range from 0 to 5 with higher scores indicative of greater cognitive impairment. A GDS of 0.5 or higher is considered a positive predictive value in establishing HIV-associated cognitive impairment (Carey et al., 2004). Accordingly, patients were categorized as either CI (GDS ≥ 0.5) or unimpaired (non-CI; GDS < 0.5) based on their performance across these six measures. The categories of CI and unimpaired were used to determine between-group differences in the expression of microRNAs.

### Alcohol Use Disorder Identification Test (AUDIT)

AUDIT is a self-administered questionaire developed by the World Health Organization to report harmful drinking behavior. Scores of 8 and above are considered as indicators of harmful drinking alcohol use.

### RNA Extraction, Quality Control and miRNA Profiling

RNA extraction and microRNA profiling were performed as previously reported (Pacifici et al., 2014; Kadri et al., 2015). RNA was obtained from 200 μl of plasma using the miRCURY RNA extraction kit (Exiqon, Woburn, MA, USA). To increase the RNA recovery, 1 μg of MS2 carrier RNA was added to each plasma sample. Eight microliter of total RNA was subjected to retro-transcription using the Universal cDNA synthesis kit (Exiqon, Woburn, MA, USA), followed by RT-qPCR using microRNA LNA primer sets (Exiqon, Woburn, MA, USA). RT-qPCR was carried out on a Roche LightCycler 480 Real-Time PCR System according to the Exiqon recommended protocol. Cycling conditions were as follows: 95°C for 10 min, 40 cycles of 15 s at 95°C, and 60 s at 60°C. Fluorescent data were converted into cycle threshold (Ct) measurements by the Roche LyghtCycler system software (Version 1.5; Roche). Quantification using second derivative maximum was further calculated with Roche LightCycler 480 software. qPCR data were analyzed in GenEx Professional 5 software (MultiD Analyses AB, Goteborg, Sweden). Degree of hemolysis was determined as the difference in Ct of miR-23a-3p (a microRNA not affected by hemolysis) and miR-451a (an indicator of hemolysis); this calculation was performed in GenEx. The amount of target microRNAs was normalized relative to the amount of reference genes, miR-23a-3p and miR-23b-3p. Fold change between the groups was calculated according to the formula 2^−ΔΔCt^. For the scatter plots in which controls are also shown we used the formula 2^−ΔCt^, which is a linear representation of raw Cts; therefore, similarly to CT values, the higher the 2^−ΔCt^, the lower the expression of the miRNA.

### Hollingshead Index

It is an index of social position developed by Hollingshead (1957) and is based on education and occupation according to the formula: (years of education × 4) + (occupation scale score × 7). The index score ranges from 11 (best social position) to 77 (lowest social position). Five classes can be formed according to the social scores: class I (11–17), class II (18–27, class III (28–43), class IV (44–60) and class V (61–77).

### Statistical Analyses

The selected miRNAs were normalized (ΔCt = Ct_miR_ − Ct_ref\gene_) relative to the amount of reference genes, miR-23a-3p and miR-23b-3p. The normalized miRNA levels (ΔCt) were used for further analyses. We considered two miRNA measures: single miRNAs and miRNA pairs. We evaluated the associations between these miRNA measures and two outcomes (cognitive impairment (yes/no) and AUD (yes/no)) using the two-sided Mann-Whitney tests. Fold change of the two study groups was calculated according to the formula 2^−ΔΔCt^, where ΔΔCt = ΔCt_yes-group_ − ΔCt_no-group_. A fold change above 2 was considered upregulatation of the miRNA, and below 0.5 was considered downregulated. For a more intuitive representation of downregulated miRNAs (fold change < 0.5) we used the negative value of the reciprocal (i.e., a fold change of 0.1 would be equal to −10; Eletto et al., 2008; Pacifici et al., 2013; Kadri et al., 2015). For each sample, miRNA pairs were calculated using the formula ΔCt_miRx_ − ΔCt_miRy_ and this difference is represented as miR_x_/miR_y_ as previously reported (Hennessey et al., 2012; Sheinerman et al., 2012, 2013a,b; Sheinerman and Umansky, 2013; Kadri et al., 2015). For the 71 miRNAs screened, each sample resulted in 2485 (^71^C_2_) miRNA pairs. Each miRNA pair from one group (i.e., CI) was compared with the same miRNA pair from the other group (i.e., non-CI) using the two-sided Mann-Whitney test. For multiple comparison justification, Bonferroni correction was applied to determine statistical significance for single miRNAs and miRNA pairs. The related Bonferroni criteria were as follow: at LSUHSC we screened 71 miRNAs (adjusted *P* < 0.001) and 2485 miRNA pairs (*P* < 0.000020); at CHARTER we screened 21 miRNAs (*P* < 0.002) and 171 miRNA pairs (*P* < 0.00029). Spearman correlations were applied to evaluate correlation bewteen miRNA levels. The descrimination level of these miRNA markers was assessed by estimating the area under the Receiver Operating Characteristic curve (AUC), sensitivity and specificity. Statistical analyses were performed using the GenEx Professional and GraphPad Prism 5 software.

### Study Approval

All studies were performed with approval from the LSUHSC Institutional Review Board (IRB #8786) and in conjunction with national guidelines for protection of patient confidentiality and safety.

## Results

### Patient Characteristics and Medical Parameters

For validation studies, we have utilized plasma from newly enrolled patients at LSUHSC and patients enrolled in the CHARTER program. Patients at LSUHSC (*n* = 66) had characteristics similar to those previously published (Kadri et al., 2015), except that AUD identification test (AUDIT) was administered to the newly recruited patients. AUDIT scores were assigned based on self-reporting drinking behaviors (Table 1). Patients enrolled in the CHARTER program (*n* = 70) were all AUD negative (Table 2). Patients with neurodegenerative disorders or brain injuries were excluded from the study. Major differences in the two patients’ populations consisted in a higher percentage of African-Americans at LSUHSC (89.5%) compared to CHARTER (55.7%); higher number of females (41%) at LSUHSC compared to CHARTER (18.6%); 14% of patients at LSUHSC had equal or less than 8 years of school, while at CHARTER all patients had more than 8 years of education.

### Validation at LSUHSC and Effect of AUD on miRNAs Associated with Cognitive Impairment

Alcohol abuse is highly prevalent among PLWHA and we sought to investigate whether plasma miRNAs could associate with AUD and whether alcohol consumption could be a confounding factor for the miRNA biomarkers associated with cognitive impairment in this patient’s population. To this end, we selected 91 miRNAs and 66 plasma samples as detailed in Table 1. The selection of miRNAs was based on the following criteria: (1) our previous study in which we have identified miRNAs and miRNA pairs associated with cognitive impairments in PLWHA at LSUHSC. This list included miRNAs that were detected in the array performed in the pilot study, but that were discarded because of the presence of more promising miRNAs; and (2) miRNAs with a validated function in alcohol abuse disorders, such as alcohol dependence and tolerance. Not all miRNAs were reproducibly detected across the samples and therefore they were discarded; the remaining 73 miRNAs (listed in Supplementary Table S1) were considered for further analysis. As we reported for the set of plasma samples used in the pilot study (Kadri et al., 2015), also in this new set of samples miR-23a-3p and miR-23b-3p were uniformly expressed across all samples and were used to normalize qPCR data. We grouped plasma samples in AUD (*n* = 27; AUDIT score ≥8) and non-AUD (*n* = 39; AUDIT score < 8), with each group containing patients with and without cognitive impairment as specified in Table 1.

**Figure 1:**
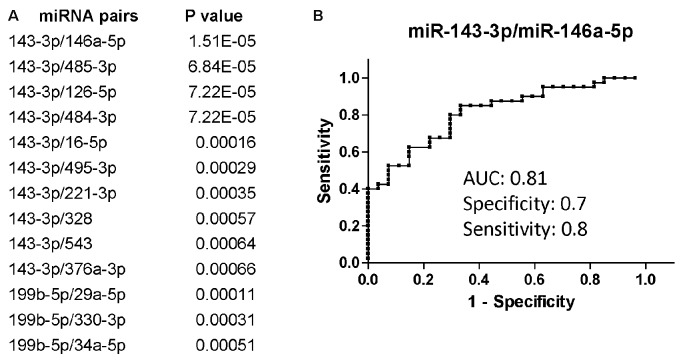
Differentially regulated miRNAs and miRNA pairs in persons living with HIV-1/AIDS (PLWHA) with alcohol use disorder (AUD) at LSU Health Sciences Center (LSUHSC). **(A)** miRNA pairs discriminating subjects with AUD (*n* = 27) and without AUD (*n* = 39). **(B)** Receiver Operator Characteristic (ROC) curve for the miRNA pair that best associated with AUD. AUC, area under the curve.

Overall, miR-143-3p and miR-199b-5p were the miRNAs that better discriminated PLWHA with AUD from those without, with a fold-change decrease of −2.09 (*p* = 0.0083) and −2.02 (*p* = 0.012), respectively (data not shown). When we evaluated the diagnostic power of those miRNAs through the Receiver Operator Characteristics (ROC) analysis the Area under the Curve (AUC) was 0.64 for miR-143-3p and 0.7 for miR-199b-5p. Those two miRNAs performed better as diagnostic markers in the miRNA pair analysis (Figure 1A) with the combination of miR-143-3p with miR-146a-5p, miR-485-3p, miR-126-5p or miR-484-3p being the best in distinguishing the two groups of AUD and non-AUD patients. All four top miRNA pairs shared similar AUC, specificity and sensitivity. Figure 1B shows the ROC analysis for the pair miR-143-3p/miR-146a-5p as a representative plot for the top four miRNA pairs. Next, we determined a possible correlation between miRNAs and the AUDIT score. We utilized Spearman’s correlation analysis and evaluated possible association between AUD scores and miRNAs or miRNA pairs. MiR-143-3p (*r* = 0.41, *P* = 0.0007) and miR-199b-5p (*r* = 0.28, *P* = 0.042) positively correlated with AUDIT scores, while miR-485-3p correlated negatively with AUDIT scores (*r* = −0.27, *p* = 0.029). The same statistical analysis was performed for the miRNA pairs that best discriminated AUD from non-AUD patients (Figure 1A). The pair 143-3p/146a-5p had the best coefficient of correlation of 0.62 (*P* < 0.0001), followed by 143-3p paired with 126-5p, 16-5p or 484-3p (r ranging from 0.51 to 0.56, *P* < 0.0001). Except for the pair 199b-5p/29a-3p that showed no correlation, the remaining pairs had negative r, suggesting an inverse correlation with the AUDIT score for those pairs. The best negative correlations had a coefficient r of −0.54 and −0.5 for the pairs 143-3p/485-3p and 143-3p/495-5p, respectively (*P* < 0.0001 for both pairs).

**Figure 2:**
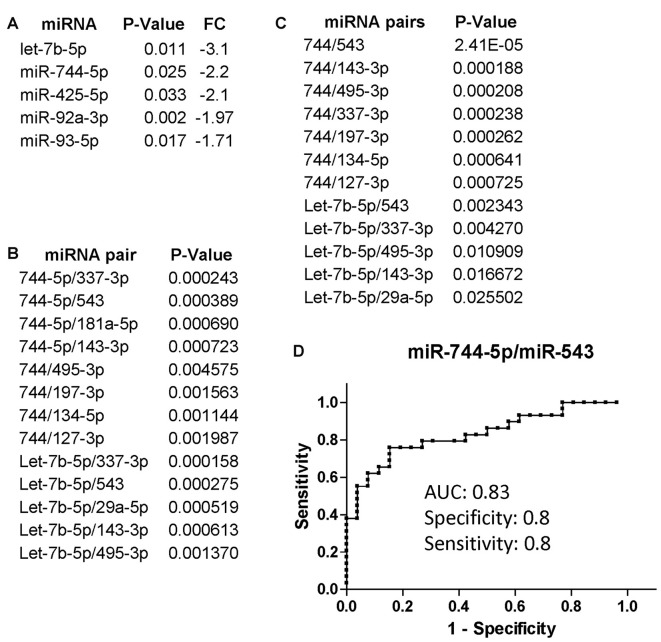
miRNA and miRNA pairs that discriminated cognitively impaired (CI) PLWHA from non-impaired at LSUHSC. List of differentially regulated miRNAs **(A)** and miRNA pairs **(B)** in CI vs. non-impaired patients. FC, fold change. Note that downregulated miRNAs (fold change < 0.5) are represented as the negative value of the reciprocal. **(C)** miRNA pairs that better discriminated CI (*n* = 29) from non-impaired (*n* = 25) subjects, after removing subjects with severe AUD (AUDIT score ≥ 18). **(D)** ROC analysis of the miRNA pair miR-744-5p/miR-543. AUC, area under the curve.

We additionally evaluated changes in miRNA expression of CI (*n* = 34 of which 22 AUD and 12 without AUD) patients compared to non-CI (*n* = 32 of which 17 AUD and 15 without AUD) and found that miR-744-5p and let-7b-5p were the most downregulated in CI patients compared to non-CI (Figure 2A). Figure 2B shows the list of miRNA pairs that better discriminated CI from non-CI patients in this set of plasma samples. Of note, the two pairs, miR-744-5p/miR-495-3p and let-7b-5p/miR-495-3p, were previously found discriminating CI from non-CI in our pilot study (Kadri et al., 2015). Finally, we further analyzed the groups of CI and non-CI after removing AUD patients with an AUDIT score of 18 and above; 5 were removed from the CI group (*n* = 29) and 7 from the non-CI group (*n* = 25). The results shown in Figure 2C indicate that miR-744 is negatively affected by AUD, since removal of plasma samples from patients with severe AUD resulted in a better *P*-value (compare Figures 2B,C) in discriminating CI from non-CI groups. Conversely, the association of let-7b-5p with cognitive impairment appears to be stronger in the presence of severe AUD, as *P*-values are about 10 times higher in Figure 2C compared to Figure 2B. The potential of miR-744-5p/miR-543 in discriminating CI from non-CI patients was evaluated through ROC analysis and results in Figure 2D show a good AUC of 0.83 and equally good sensitivity and specificity (both 0.8).

### CHARTER Cohort: Validation of Reference Genes miR-23a-3p and miR-23b-3p

**Figure 3:**
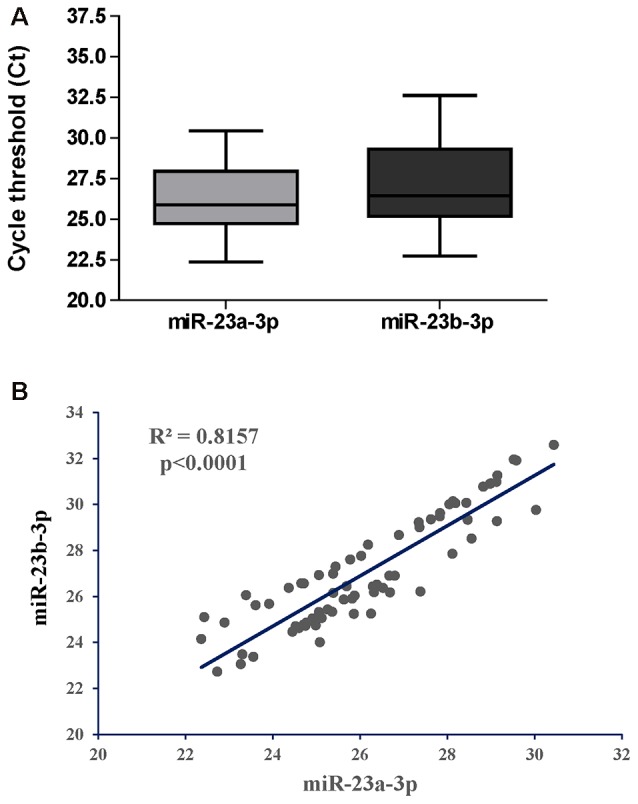
Performance of miR-23a-3p and miR-23b-3p as reference genes in the group of PLWHA from the CHARTER study. **(A)** Relative expression (raw Cts) of miR-23a-3p and miR-23b-3p across all samples (*n* = 70). **(B)** Graphical representation of Pearson’s correlation analysis between the two miRNAs.

We first asked if miR-23a-3p and miR-23b-3p were uniformly expressed throughout the CHARTER plasma samples and if they could serve as reference genes as they did for the samples from LSUHSC (Kadri et al., 2015). Figure 3A shows box plots representing Ct values of miR-23a-3p and miR-23b-3p across all 70 samples with an average of 26.1 and 27.1, respectively. In addition, the Ct values of the same two miRNAs had a strong positive Pearson’s correlation (*r* = 0.8157, Figure 3B). Both, Ct values and coefficient of correlation r were comparable to those found in our previous study using LSUHSC samples (Kadri et al., 2015). Therefore, miR-23a-3p and miR-23b-3p were used as reference genes to normalize data in the present study.

### Differentially Regulated miRNAs at CHARTER

**Figure 4:**
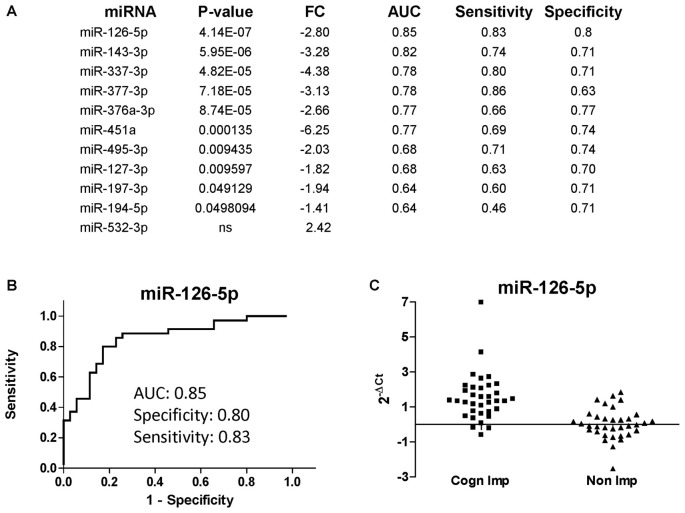
miRNAs discriminating CI from non-impaired PLWHA at CHARTER. **(A)** List of miRNAs associated with cognitive impairments (CI, *n* = 35; non-CI, *n* = 35), ordered according to the best *P*-value. Relative expression (FC, fold change), AUC, sensitivity and specificity are indicated for each miRNA. Note that miR-532-3p was the only upregulated miRNAs, but the difference was not statistically significant. **(B)** ROC analysis of the best miRNA biomarker, miR-126-5p. **(C)** Scatter plot showing relative expression of miR-126-5p in the two groups of CI and non-impaired patients.

The list of miRNAs to be profiled was based on our previous study performed at LSUHSC (Kadri et al., 2015) and consisted of 21 miRNAs (Table 3). UNISP6 was used as an internal control for plate-to-plate variations and for cDNA quality, while miR-23a-3p and miR-23b-3p were used for normalization. MiR-126-5p, miR-143-3p, and miR-543 were originally discarded in our pilot study because of the presence of more promising miRNAs in the array data (Kadri et al., 2015). However, in revising the arrays we decided to include these miRNAs in the present study. Figure 4A shows the list of differentially regulated miRNAs in CI individuals compared to non-impaired ordered according to the best (lowest) *P*-value, as determined by Mann-Whitney tests. Only one miRNA, miR-532-3p, was upregulated in CI individuals, however this difference was not statistically significant. The most downregulated miRNAs were miR-451 and miR-337-3p (6.25 and 4.38 folds, respectively), followed by miR-143-3p (3.28), miR-377-3p, miR-126-5p (2.80) and miR-376a-3p (2.66). The diagnostic performance of the differentially regulated miRNAs was evaluated through ROC analyses and results, represented by the area under the curve (AUC), sensitivity and specificity, are shown in Figure 4A (right lanes). MiR-126-5p was the miRNA with the best AUC (0.85), sensitivity (0.83) and specificity (0.80), as shown in Figure 4B. Relative expression of miR-126-5p in the two groups of patients, CI and non-impaired, is represented in the scatter plots in Figure 4C.

**Table 3:** List of 22 miRNAs used for the validation at CHARTER.

UniSP6	hsa-miR-23b-3p
hsa-miR-126-5p	hsa-miR-337-3p
hsa-miR-127-3p	hsa-miR-376a-3p
hsa-miR-143-3p	hsa-miR-377-3p
hsa-miR-146a-5p	hsa-miR-451a
hsa-miR-151a-5p	hsa-miR-487b
hsa-miR-16-5p	hsa-miR-495-3p
hsa-miR-194-5p	hsa-miR-532-3p
hsa-miR-197-3p	hsa-miR-543
hsa-miR-221-3p	hsa-miR-744-5p
hsa-miR-23a-3p	hsa-let-7b-5p

**Figure 5:**
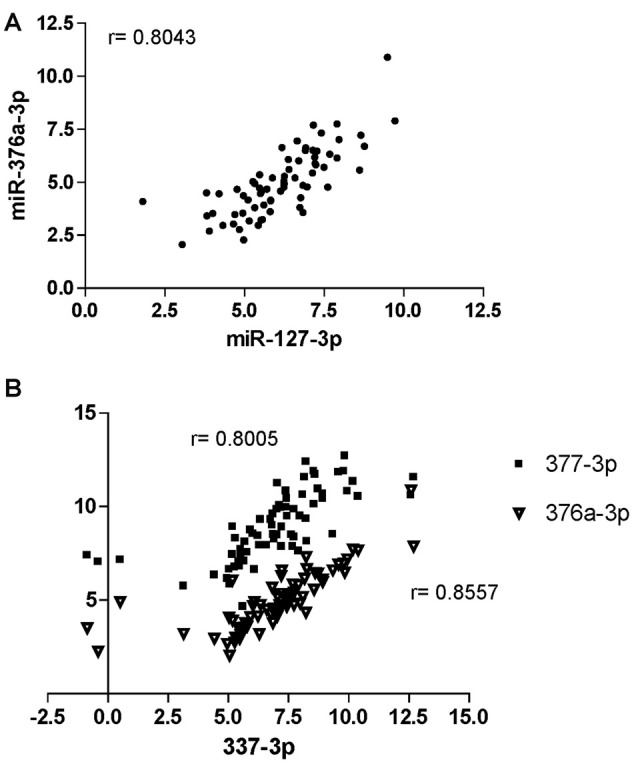
Correlation analysis of miRNAs at CHARTER. Positive correlation exists between miR-376a-3p and miR-127-3p **(A)**, and miR-337-3p with both miR-377-3p and 376a-3p **(B)**. Correlation factors (r) are indicated for each pair.

Spearman’s correlation analysis revealed statistically significant (*P* < 0.05) monotonic relationships between several miRNAs (data not shown). The best correlations (*r* > 0.8) were found for the pairs miR-127-3p/miR-376a-3p (*r* = 0.8043; Figure 5A) and miR-337-3p with either miR-376a-3p (*r* = 0.8557) or miR-377-3p (*r* = 0.8005; Figure 5B).

### miRNA Pairs Distinguishing PLWHA with CI from Those without CI

**Figure 6:**
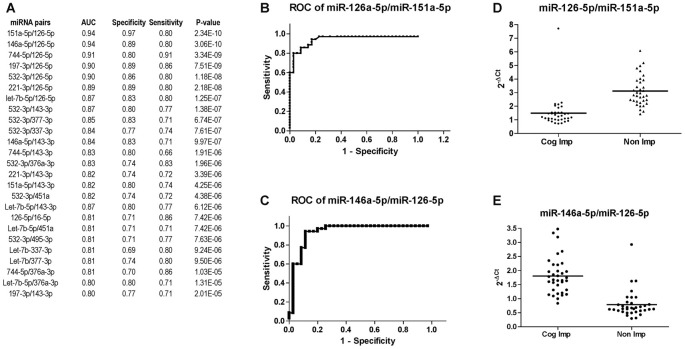
Diagnostic power of miR-126-3p is increased when paired with miR-151a-5p or miR-146a-5p. **(A)** List of miRNA pairs that distinguished CI (*n* = 35) from non-impaired (*n* = 35) patients at CHARTER, ordered according to the best *P*-value. AUC, sensitivity and specificity are also reported in the list. ROC analysis of the two miRNA pairs that best discriminated the two groups, miR-151a-5p/miR-126a-5p and miR-146a-5p/miR-126-5p, are shown in **(B,C)**, respectively; their relative expression levels are plotted in **(D,E)**, respectively.

miRNA expression analysis was further performed using the miRNA pairing approach (Boeri et al., 2011; Sheinerman et al., 2012, 2013a,b; Kadri et al., 2015; Sharova et al., 2016). The list of the best miRNA pairs that discriminated the group of CI PLWHA from those non-impaired is shown in Figure 6A. In the list, miRNA pairs are ranked according to their P-values, as determined by Mann-Whitney test. Next, ROC analysis was also performed for the miRNA pairs and those with an AUC greater or equal to 0.8 are shown in Figure 6A, together with sensitivity and specificity. A graphical representation of the best two miRNA pairs, miR-126-5p/miR-151a-5p and miR-126-5p/miR-146a-5p, is depicted in Figures 6B,C. AUC (0.94) and sensitivity (0.80) were the same for those miRNA pairs while specificity was higher for the pair 126-5p/miR-151a-5p (0.97) compared to the one of miR-126-5p/miR-146a-5p (0.89). Relative expression of the two miRNA pairs is plotted in Figures 6D,E.

### Effect of BMI on miRNA Biomarkers for CI

**Figure 7:**
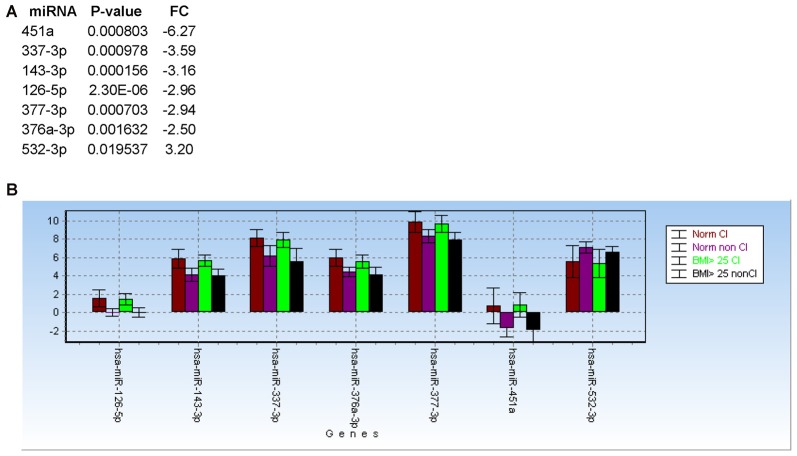
Body max indexes (BMI) has no effect on miRNA biomarkers for cognitive impairments in PLWHA. **(A)** List of miRNAs associated with cognitive impairment after removal of samples from patients with obesity (BMI ≥ 25). **(B)** Bar graph showing relative expression (expressed as 2^−ΔCt^) of the same miRNAs in the indicated groups of patients. Norm: normal weight. Norm CI, *n* = 14; norm non-CI, *n* = 15; BMI ≥ 25 CI, *n* = 19; BMI ≥ 25, *n* = 18.

Based on body max indexes (BMI) we grouped patients at CHARTER into three categories: normal weight (BMI < 25), overweight (BMI between 25 and 30) and obese (BMI > 30). This classification was distributed between CI and non-impaired (non-CI) patients as detailed in Table 2. When we analyzed patients with normal weight compared to patients with BMI ≥ 25, regardless of the cognitive status, none of the 21 miRNAs examined showed a statistically significant difference between these two groups, suggesting the miRNA we analyzed are not associated with BMI. Furthermore, when we removed samples from patients with BMI ≥ 25, miRNAs 126-5p, 143-3p, 451a, 337-3p, 377-3p, 376a-3p and 532-3p still discriminated CI from non-impaired patients, with miR-126-5p being the best (Figure 7A; see also Figure 4A). In fact, the trend of expression of these selected miRNAs was comparable in controls CI and non-CI, and BMI ≥ 25 CI compared to BMI ≥ 25 non-CI (Figure 7B).

## Discussion

We have previously identified a plasma miRNA signature that distinguish CI from non-impaired PLWHA in care at LSUHSC HOP clinic. While we did not considered alcohol use in the pilot study, the two groups of CI and non-impaired patients likely contained patients with AUD. Therefore, we have investigated the miRNA biomarker signature in the context of AUD with and without cognitive impairments in newly recruited PLWHA at LSUHSC HOP clinic. We found miRNAs (miR-143-3p and miR-199b-5p) and miRNA pairs associated with AUD (Figure 1).

In order to investigate the effect of alcohol misuse in the miRNA biomarkers for cognitive impairment, we removed from the analysis patients with an AUDIT score equal or above 18. Among the miRNAs that distinguished CI PLWHA from controls, we found miR-744-5p/miR-495-3p and let-7b-5p/miR-495-3p, two pairs that confirmed our previous pilot study (Kadri et al., 2015). Interestingly, one miRNA, let-7b-5p, correlated strongly with cognitive impairments in the presence of severe AUD. Notably, extracellular let-7b activates the RNA-sensing Tall-like Receptor 7 (TLR7) and induces neurodegeneration through neuronal TLR7 (Lehmann et al., 2012). In addition, ethanol induces hippocampal expression of let-7b-5p and TLR7, which in turn triggers the release of let-7b-5p into microglia-derived microvesicles, contributing to ethanol induced neuroimmune pathology (Coleman et al., 2017).

We also asked how the miRNA signature for cognitive impairment identified in our initial study would perform in an independent cohort of patients. To this end, we obtained 70 (35 CI and 35 controls; all AUD negative) plasma samples from the CHARTER study and we measured changes in the expression of 21 miRNAs. This list of miRNAs included miR-126-5p, miR-143-3p and miR-543, three miRNAs that were not tested in the pilot study because of the presence of more promising miRNAs, but were found to be good biomarkers in the LSUHSC cohort of patients (Figures 1A, 2B,C). Previously, we found that miR-23a-3p and miR-23b-3p were uniformly expressed across all plasma samples and they could serve as reference genes (Kadri et al., 2015); here, we confirmed their performance as reference genes in the new set of plasma samples at LSUHSC as well as in the samples from the CHARTER study (Figure 3). Since optimal normalization is a critical step for miRNA-related studies, this important finding could allow better cross-cohorts comparison, at least for PLWHA.

In our study, we did not find any association between the 21 selected miRNAs and BMI. In addition, BMI did not seem to affect the selected miRNAs and/or their power in discriminating CI from non-CI subjects. However, the information about BMI was not available for the subjects enrolled at LSUHSC and we could not validate these data. Therefore, BMI, or other indicators of obesity, could be considered in future studies aimed at evaluating the impact of obesity on the miRNAs associated with cognitive deficits.

**Figure 8:**
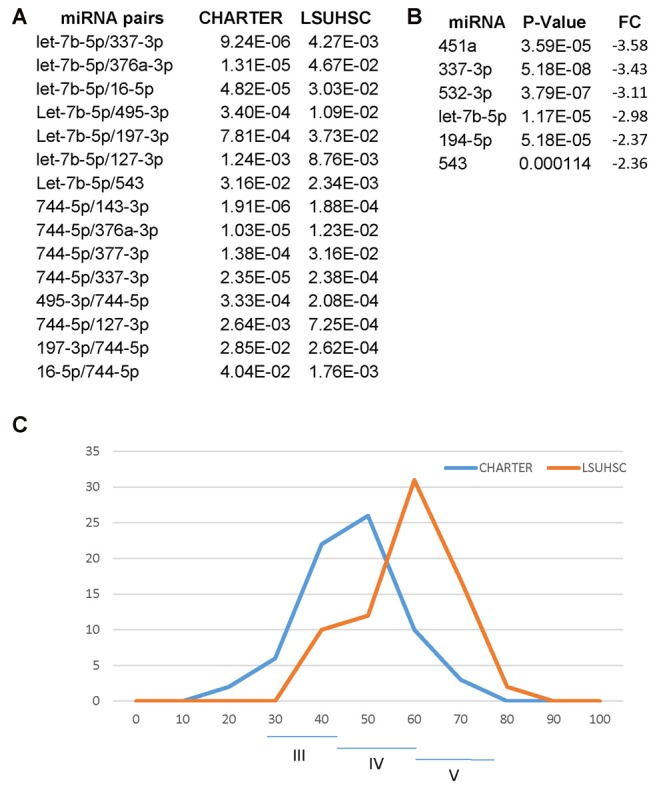
Differences and similarities between miRNA and miRNA pairs at LSUHSC and at CHARTER. **(A)** *P*-value comparison between miRNA pairs biomarkers for cognitive impairments common to LSUHSC and CHARTER. **(B)** List of miRNAs downregulated at LSUHSC compared to CHARTER. Fold change (FC) and *P*-values are shown. **(C)** Graph representing the normal distribution of the socioeconomic index (Hollingshead index) at CHARTER and LSUHSC. The higher the score (max 77), the lower the ranking in social position. Classes III (scores 24–43), IV (scores 44–60) and V (61–77) are indicated.

One goal of the present study was to confirm the miRNA signature for cognitive impairment in an independent cohort of PLWHA and we found 15 miRNA pairs that distinguished CI patients in both sites, LSUHSC and CHARTER (Figure 8A). Interestingly, we also found differences between LSUHSC and the CHARTER samples. We found that some of the miRNAs associated with cognitive impairment in the CHARTER group (Figure 4A) were downregulated at LSUHSC compared to CHARTER samples (451a, 337-3p, 532-3p, 194-5p and 543; Figure 8B). While we cannot explain the reason for a 2-3-fold downregulation of the miRNAs in the LSUHSC cohort, we looked at the characteristics of the two cohorts of patients, particularly at the socio-economic status, as poverty is a prevalent factor among PLWHA in New Orleans area (Louisiana STD/HIV quarterly report 2016, Vol. 14). We noticed that the patients at LSUHSC were all from New Orleans area, while in the CHARTER group only 15.5% were from southern US and the remaining 84.5% from northern US (53% northeast and 31.5% northwest). Based on the Hollingshead two-factors index (Hollingshead, 1957), which measures the socioeconomic status on the basis of education and occupation, we also found that the frequency of distribution of this index is shifted toward a lower socioeconomic status (higher index values) in LSUHSC patients compared to CHARTER (Figure 8C). Nevertheless, despite the differences in social status, and perhaps conditions linked to it, between LSU and CHARTER, we still found common miRNA pairs discriminating CI from non-impaired patients. Therefore, the data presented here suggest that our novel approach using miRNA pairs could robustly serve as biomarkers for CI regardless of other conditions, including behavioral and social environmental. However, there are several limitations in our study worth mentioning. One is related to the complexity of the disease and the many factors potentially affecting the circulating miRNAs. We have only considered substance abuse but factors such as co-infections, depression and suicidal behaviors are also relatively common problems in PLWHA (Nanni et al., 2015; Tao et al., 2017; Vreeman et al., 2017) and they could likely have an impact on the miRNA neuro-biomarkers for cognitive impairments. Another consideration is related to the intrinsic nature of miRNAs and their key function in brain development and neuronal fitness (Sun et al., 2013; Radhakrishnan and Alwin Prem Anand, 2016; Roese-Koerner et al., 2017; Yang et al., 2017). Similarly to any other gene, miRNAs are subjected to sequence polymorphism such as single nucleotide polymorphisms (SNPs) that could change their function and that were not considered in this study. Since we are detecting only mature miRNAs, if a SNP affecting the expression of the miRNA would be prevalent in the general population that miRNA would have not been detected by qPCR and therefore excluded from the study. Nevertheless, it could be interesting in the future to study SNPs affecting miRNAs and/or their targets when looking at the potential function of miRNA neuro-biomarkers in the brain pathology.

Finally, we would like to point out several important observations that allowed us to understand better how to approach the search for biomarkers in a complex population such as PLWHA. We started with a pilot project using an array approach, which allowed us to find putative biomarkers for cognitive impairments. Such an approach however had the important limitation of masking some miRNAs that we dismissed because they were not strongly deregulated in the array, but that performed much better in the individual PCRs (this work). Importantly, we found that AUD can affect miRNA biomarkers associated with cognitive impairments and that the identification of such markers, such as let-7b-5p, could unravel potential mechanisms of neuropathogenesis. With the identification of miRNA biomarkers for cognitive decline in PLWHA our study serves as a basis for the development of non-invasive and novel diagnostic tools for monitoring cognitive deficits and patterns of substance use in PLWHA. Successful definition of a sensitive and specific neuro-biomarker panel will allow for implementation of cognitive, behavioral, and pharmacologic strategies to reduce progression of neurological decline and substance use. This approach will ultimately contribute to improving cART adherence and quality of life, particularly among high-risk, underserved HIV-positive individuals.

## Author Contributions

DW, DJ and AL performed the experiments; H-YL performed statistical analysis; ALP administered neurocognitive assessment to patients; CHP coordinated the patients at LSUHSC HOP clinic; PEM edited the manuscript; FP designed the experiments and wrote the manuscript.

## Conflict of Interest Statement

The authors declare that the research was conducted in the absence of any commercial or financial relationships that could be construed as a potential conflict of interest.
