# Supplementary material for: A miRNA Signature for Cognitive Deficits and Alcohol Use Disorder in Persons Living with HIV/AIDS
Source: Front Mol Neurosci. 2017 Nov 15;10:385. doi: 10.3389/fnmol.2017.00385 (PMC5694774; doi:10.3389/fnmol.2017.00385)
Supplement: Supplementary file 1 [file Table_1.docx]

*Supplementary Material*

**A miRNA signature for cognitive deficits and alcohol use disorder in persons living with HIV/AIDS**

Dorota Wyczechowska^1, 2^, Hui-Yi Lin^1,3^, Andrea LaPlante^1^, Duane Jeansonne^1^, Adam Lassak^1, 2^, Christopher Parsons^1, 2,5^, Patricia E Molina^1, 4,6^, Francesca Peruzzi^1, 2, 5,6 *^

*Correspondence: Francesca Peruzzi, fperuz@lsuhsc.edu

Supplementary Tables:

**Table 1.** List of 73 miRNAs used for the LSUHSC study.

| \| hsa-miR-103a-3p \| \| hsa-miR-221-3p \| \| hsa-miR-377-3p \| \| --- \| --- \| --- \| --- \| --- \| \| hsa-miR-106a-3p \| \| hsa-miR-223-3p \| \| hsa-miR-382-5p \| \| hsa-miR-125a-5p \| \| hsa-miR-22-3p \| \| hsa-miR-409-3p \| \| hsa-miR-125b-5p \| \| hsa-miR-22-5p \| \| hsa-miR-425-5p \| \| hsa-miR-126-5p \| \| hsa-miR-26a-5p \| \| hsa-miR-431-5p \| \| hsa-miR-127-3p \| \| hsa-miR-29a-3p \| \| hsa-miR-433-3p \| \| hsa-miR-130a-3p \| \| hsa-miR-29a-5p \| \| hsa-miR-451a \| \| hsa-miR-132-3p \| \| hsa-miR-30b-5p \| \| hsa-miR-484 \| \| hsa-miR-133b \| \| hsa-miR-323a-3p \| \| hsa-miR-485-3p \| \| hsa-miR-134-5p \| \| hsa-miR-326 \| \| hsa-miR-487b \| \| hsa-miR-139-5p \| \| hsa-miR-328 \| \| hsa-miR-491-5p \| \| hsa-miR-143-3p \| \| hsa-miR-329-3p \| \| hsa-miR-495-3p \| \| hsa-miR-146a-5p \| \| hsa-miR-330-3p \| \| hsa-miR-532-3p \| \| hsa-miR-148a-3p \| \| hsa-miR-335-5p \| \| hsa-miR-543 \| \| hsa-miR-151a-3p \| \| hsa-miR-337-3p \| \| hsa-miR-744-5p \| \| hsa-miR-151a-5p \| \| hsa-miR-338-3p \| \| hsa-miR-92a-3p \| \| hsa-miR-154-5p \| \| hsa-miR-34a-5p \| \| hsa-miR-92b-3p \| \| hsa-miR-16-5p \| \| hsa-miR-362-3p \| \| hsa-miR-93-5p \| \| hsa-miR-181a-5p \| \| hsa-miR-369-3p \| \| hsa-let-7b-3p \| \| hsa-miR-181b-5p \| \| hsa-miR-370-3p \| \| hsa-let-7b-5p \| \| hsa-miR-194-5p \| \| hsa-miR-374b-5p \| \| hsa-let-7c-5p \| \| hsa-miR-197-3p \| \| hsa-miR-376a-3p \| \| hsa-let-7d-3p \| \| hsa-miR-199a-5p \| \| hsa-miR-376b-3p \| \| hsa-let-7d-5p \| \| hsa-miR-199b-5p \| \| hsa-miR-376c-3p \| \| hsa-miR-23a-3p \| \|  \|  \|  \|  \| hsa-miR-23b-3p \| |  |
| --- | --- | --- | --- | --- | --- | --- | --- | --- | --- | --- | --- | --- | --- | --- | --- | --- | --- | --- | --- | --- | --- | --- | --- | --- | --- | --- | --- | --- | --- | --- | --- | --- | --- | --- | --- | --- | --- | --- | --- | --- | --- | --- | --- | --- | --- | --- | --- | --- | --- | --- | --- | --- | --- | --- | --- | --- | --- | --- | --- | --- | --- | --- | --- | --- | --- | --- | --- | --- | --- | --- | --- | --- | --- | --- | --- | --- | --- | --- | --- | --- | --- | --- | --- | --- | --- | --- | --- | --- | --- | --- | --- | --- | --- | --- | --- | --- | --- | --- | --- | --- | --- | --- | --- | --- | --- | --- | --- | --- | --- | --- | --- | --- | --- | --- | --- | --- | --- | --- | --- | --- | --- | --- | --- | --- | --- | --- |
